# Clinical Confirmation of High Potencies

**Published:** 1888-01

**Authors:** B. L. B. Baylies

**Affiliations:** Brooklyn, N. Y.


### CLINICAL CONFIRMATION OF HIGH POTENCIES.

B. L. B. Baylies, M. D., Brooklyn, N. Y.

The following represents the clinical confirmation of some of the characteristics of remedies applied in the treatment during more than four years of a patient who had for a long period suffered with uterine disorders. The lady, about forty years of age, fat and lymphatic, but at the same time highly nervous, had complete antero-version of the uterus, with chronic metritis, and had, previously to my attendance, been treated allopathically, especially for inflammation of the ovaries and for abscess of the left ovary, which discharged through the vagina. She was, when I first saw her, almost wholly unable to walk, in consequence of the malposition of the uterus, the fundus of which was firmly impacted behind the pubis, the cervix in the hollow of the sacrum. I do not attempt to demonstrate the benefit of any particular course of treatment of uterine displacements illustrated in the management of this case, but merely to state the several groups of symptoms, whether casual or necessarily connected with the condition, which were always relieved by the remedies specified. I will premise that some mechanical expedients for relief were employed. Among pessaries the watch spring pessary, affording considerable ease for weeks at a time, and Fraser's cup-shaped pessary, applied twice, and worn several weeks at each time, its removal being necessitated by abrasion of the cervix and post-pubic mucous membrane. But this did not happen till the instrument had been worn several weeks, during which the patient enjoyed great comfort and cheerfulness, and was entirely relieved of uterine tenesmus, sighing respiration, and all the symptoms of impaired function of the bladder and rectum, caused by the pressure of the uterus; but after its removal, not the least improvement was evident; the displacement continued as before, the treatment only temporarily beneficial and not thoroughly cleanly. Thereafter, except the elastic abdominal belt, all mechanical treatment was abandoned, and medicine, with occasionally the knee-elbow position, alone employed.

Burning sensation in the epigastrium, with burning and drawing in the left ovary, was removed by Ammonium carbon.$^{200}$

2. Feeling as if the left arm and hand were wooden. Nitrum$^{20m}$, Fincke.

3. Giddiness, numbness in the hands, pricking pains of the arms and shoulders; inward fluttering; dry cough, with sharp pains in the stomach, rising of frothy mucus with sense of constriction of the throat. Cocculus indicus$^{26m}$, Fincke.

4. Swelling and sore pain in the left ovary, burning and stiff and benumbed feeling about the left hip. Lachesis $^{40m}$, Fincke.

5. Sharp pricking feeling in the right ovary, with empty sinking sensation at the epigastrium and disposition to weep. Ignatia$^{40m}$, Fincke.

6. Bearing down, cutting or clutching pain in the uterus. Belladonna$^{6th,\ 200th,\ and\ 97m}$, Fincke.

7. Fluttering, bounding, quivering, internal and external, muscular, with nervous and lachrymose mood, and sensation of heat and weakness of the right eye, and as of a scum upon the ball, which might be rubbed off; feeling as of something alive in the abdomen ; with hemorrhage, even when moderate, tending to syncope ; with discharge of black stringy clots. Crocus sativus$^{20m\ and\ 40m}$, Fincke.

8. Hysteric nervousness, sleeplessness, trembling, disposition to start or get out of bed with wild alarm, retention of urine. Hyosciamus$^{200th\ and\ 3m}$, Fincke.

9. Viscid leucorrhœa, with soreness and smarting of the vagina and vulva. Hydrastis$^{200}$, and locally.

10. Pressing pain in the forehead from without inward, gradually increasing and decreasing; flushes of heat with burning in the face, interrupted by chilliness ; ill-humor, aversion to food on account of feeling low-spirited, pressing downward during the menses, and discharge of dark clotted blood. Patient says the uterus "feels hard," *i. e.*, subjectively; cramp pain in the legs, ameliorated by heat. She sleeps with the arms over the head, knees drawn up ; shortness of breath as if the chest were constricted, and palpitation. Platina$^{200}$, Lehrmann.

11. Urine thick, cloudy, containing white mucus ; stains the linen yellow ; pain extending from the region of the right kidney along the ureter ; burning pain under the skin in the left side of the abdomen ; sticking and dragging pain at times ; burning and drawing sensation in the right inguinal region, compared by the patient to the crisping of burnt leather. These symptoms were removed soon after giving Berberis v.$^{8m}$, F.

12. Feeling as of a lump of mucus in the throat, and cough worse about 3 A. M. ; hollow weak feeling at the stomach, with easy eructations of gas ; rolling flatulence, and sensation as if in the revolutions of the bowels something touched the uterus and caused *sore* pain ; pricking and shooting downward and inward, from near the anterior superior spines of the ilia toward the ovaries ; retarded menses. Kali carb.$^{30m}$, F.

13. Painful and distressing sensation as of something pressing in the hollow of the sacrum, and behind the pubis; urging to stool as if diarrhœa would come on, or she must pass water; with great difficulty in emptying the bladder; dragging from the waist downward; drawing cramp pain in the calves of the legs; when mounting a step, drawing in the loins and back; sinking feeling at the navel; sighing; pressing down as if the contents of the pelvis would come into the world. Lilium tigrin.$^{30th}$. A single dose was given dry, and allowed to act without repetition for several days; for if the dose was repeated on two or three successive days the symptoms were aggravated. More enduring relief was afforded when the uterus was first replaced and a dose of Lilium given than when replaced without the use of Lilium. The 30th was borne well, while the 200th or higher was followed by aggravation.

14. Catching pain in the back and diaphragm, with gasping; cannot breathe without pain; after exposure to a draught, or too much exertion in walking, portions of the rectus abdominis of the external oblique or lumbar muscles affected with tonic contractions, hard to the touch. Petroleum$^{200}$, Lehr.

15. Constipation; dark fæces, coated with and connected by strings of mucus; pain extending from the anus to the back, continuing long after the passage; smarting at the anus, which feels cut or lacerated by the passage; languor and weakness in sacro-iliac region. Graph.$^{200}$.

16. Diarrhœa with hemorrhoids; bloated abdomen; constant threatening of the bowels to move, and sensation of heat in the rectum. Aloes$^{40m}$, L.

17. Colic, with distention, and cardialgia; sour eructations; dry cough, with burning in the throat, very troublesome at about eleven P. M. Magnesia carb.$^{20m}$, F., or $^{200}$.

18. Rawness (sensation) in the throat, extending to mid-sternum; irritation, extending from upper sternum toward upper left side of chest, with burning in the epigastrium and præcordium, and dry, barking cough; disposition to cover the mouth and warm the inhaled air. Rumex crispus$^{200}$ and higher.

19. Pain in the right knee when sitting for some time; after rising, a full feeling in the head and sensation as of a string drawn tightly around the head below the ears, stopping them up. Gelsemium nitridum$^{3m}$, F.

(The *Materia Medica* gives "sensation as of a tape around the head; sudden and temporary loss of hearing" under Gelsemium.)

The high potencies of Fincke were chiefly used, and, as a rule, produced their effects more promptly and rapidly than lower potencies, the patient often expressing herself conscious of the commencing operation of the medicine within five minutes after taking the dose, and was frequently relieved of great distress in fifteen minutes or half an hour. The displacement was not cured by the treatment, but her condition much ameliorated, so that she was enabled to walk more and better than for many years previously.

I do not mention the high potencies as a subject for controversy, believing that a practical conviction of the truth of the two primary and essential principles of Homœopathy, viz.: similarity and simplicity, *i. e.*, the single remedy, exacts the use of the least requisite dose. Individual judgment will determine for each physician that dose, but experiment will approve that the higher will often *complete* what the lower potencies have only partially effected, and demonstrate that the limitation of the molecule is not the limitation of force.

